# Prediction of esophageal and gastric histology by macroscopic diagnosis during upper endoscopy in pediatric celiac disease

**DOI:** 10.3748/wjg.v23.i4.646

**Published:** 2017-01-28

**Authors:** Erin D Boschee, Jason Y K Yap, Justine M Turner

**Affiliations:** Erin D Boschee, Department of Pediatrics, University of Alberta, Edmonton, Alberta T6G 2B7, Canada; Erin D Boschee, Edmonton Clinic Health Academy, University of Alberta Hospital, Edmonton, Alberta T6G 2B7, Canada; Jason YK Yap, Justine M Turner, Division of Pediatric Gastroenterology, Department of Pediatrics, University of Alberta, Edmonton, Alberta T6G 2B7, Canada

**Keywords:** Endoscopy, Histology, Esophagus, Gastric biopsy, Celiac disease

## Abstract

**AIM:**

To determine the sensitivity of macroscopic appearance for predicting histological diagnosis at sites other than duodenum in pediatric celiac disease (CD).

**METHODS:**

Endoscopic and histologic findings in pediatric patients undergoing upper endoscopy for first-time diagnosis of CD at Stollery Children’s Hospital from 2010-2012 were retrospectively reviewed.

**RESULTS:**

Clinical charts from 140 patients were reviewed. Esophageal and gastric biopsies were taken in 54.3% and 77.9% of patients, respectively. Endoscopic appearance was normal in the esophagus and stomach in 75% and 86.2%. Endoscopic esophageal diagnoses were eosinophilic esophagitis (EE) (11.8%), esophagitis (7.9%), glycogenic acanthosis (1.3%) and non-specific abnormalities (3.9%). Endoscopic gastric diagnoses were gastritis (8.3%), pancreatic rest (0.9%), and non-specific abnormalities (4.6%). Histology was normal in 76.3% of esophageal and 87.2% of gastric specimens. Abnormal esophageal histology was EE (10.5%), esophagitis (10.5%), glycogenic acanthosis (1.3%) and non-specific (1.3%). Gastritis was reported in 12.8% of specimens. Sensitivity and specificity of normal endoscopy for predicting normal esophageal histology was 86.2% and 61.1%, and for normal gastric histology was 87.4% and 21.4%.

**CONCLUSION:**

In the absence of macroscopic abnormalities, routine esophageal and gastric biopsy during endoscopy for pediatric CD does not identify major pathologies. These findings have cost and time saving implications for clinical practice.

**Core tip:** We performed a retrospective chart review of esophageal and gastric endoscopic and histologic findings in pediatric patients diagnosed with celiac disease (CD). Our findings suggest that, in the absence of macroscopic abnormalities, routine esophageal and gastric biopsy during upper endoscopy for pediatric CD does not identify major pathologies. The implication of limiting biopsies to the duodenum and duodenal bulb may be both cost and time-saving. Overall, the results of this study may have the ability to promote standardization and optimal resource allocation for routine diagnostic practices for pediatric CD.

## INTRODUCTION

Celiac disease (CD) is an autoimmune gluten-dependent enteropathy characterized by small intestinal inflammation and villous atrophy in genetically susceptible individuals[1]. Intestinal inflammation and activation of the immune system leads to production of autoantibodies such as anti-tissue transglutaminase (aTTG) and endomysial antibodies (EMA), which are now widely used as a key component of screening and diagnosis[2]. The preferred diagnostic approach in North America involves both serology and confirmatory upper endoscopy with small bowel biopsy. While the diagnosis of CD has increased dramatically in recent decades, with an estimated prevalence in North America of 1%, CD is still felt to be significantly underdiagnosed[1,3].

Accurate diagnosis of celiac disease requires a minimum of six duodenal biopsies; a minimum of four biopsies from the distal duodenum and one or two biopsies from the duodenal bulb are recommended[4]. Current guidelines for endoscopic and histologic diagnostic practices for CD focus on duodenal biopsies; there is no consensus over a standard approach to the upper endoscopy as a whole. Therefore, in the absence of specific guidelines practice is likely to vary regarding additional biopsies taken during endoscopy, which may be taken routinely from sites other than the duodenum even in the face of normal microscopic findings. Additional biopsies have the potential to add considerable additional cost for the pathological assessment of this common duodenal disorder. Furthermore, accumulating evidence suggests that a biopsy-avoiding approach may be accurate in select pediatric patients with CD[5-10]. This approach has not been endorsed for North America, in part given concerns over the potential for missing alternate tissue diagnoses[11]. Increasing demands and resource allocation pressure on existing endoscopy resources in Canada may mean that such an approach, if proven low risk, could be considered. However, there is a paucity of studies investigating the frequency of endoscopic and histological abnormalities in intestinal sites apart from the duodenum in pediatric patients with CD and the utility of endoscopic diagnosis in predicting tissue histology in the stomach and esophagus in CD has yet to be studied.

We therefore aimed to study the sensitivity of normal esophageal and gastric macroscopic appearance in predicting normal tissue histology at these sites in pediatric patients with CD. Our secondary goal was to report the prevalence of coexistent esophageal and gastric diagnoses in pediatric CD. We hypothesized that normal endoscopic appearance is highly predictive of normal histology in the esophagus and stomach in pediatric patients with CD, thereby obviating the need to routinely biopsy these areas in the absence of gross macroscopic abnormalities. Additionally, we hypothesized that few pediatric patients with CD would have coexistent gastrointestinal diagnoses of clinical significance, suggesting that few alternate diagnoses would be missed by a biopsy avoiding approach in intestinal sites other than the duodenum.

## MATERIALS AND METHODS

The research study was approved by the University of Alberta Human Research Ethics Board. A single researcher (Boschee ED) performed chart review and data collection. Patients were identified retrospectively from the Stollery Children’s Hospital Celiac Disease Clinic database, 2010-2012, and included following review of patient clinic charts. Criteria for inclusion were: age between 0 and 18 years at the time of biopsy; completion of an assessment by a pediatric gastroenterologist at the Stollery Children’s Hospital following referral to consider a diagnosis of celiac disease; completion of first-time diagnostic upper endoscopy and duodenal biopsy; and subsequent proven histological diagnosis of CD. All patients diagnosed with CD had duodenal histological classification of Marsh grade 2 or 3, as per current NASPGHAN guidelines[12]. Patients following a gluten free diet at the time of endoscopy and biopsy were excluded, as were those with a prior diagnosis of CD or with non-confirmatory histology (Marsh grade 0 or 1).

Patient demographic information collected included age at the time of biopsy, as well as gender, growth parameters and presenting symptoms (if any). When available, serological test results including serum aTTG, IgA and EMA prior to endoscopy and biopsy were recorded. Endoscopy and histology reports were reviewed for the reported endoscopic and histologic diagnoses in the esophagus and stomach.

The statistical analyses of this study were performed by biostatisticians, Dr. Maryna Yaskina and Sung Hyun Kang, of the Women and Children’s Health Research Institute. Statistical analyses were performed using SPSS 23 and R version 3.2.3 software. Continuous variables were expressed as means, and statistical significance was defined by alpha less than 0.05. Sensitivity, specificity, positive and negative predictive values, and positive and negative likelihood ratios were calculated using a 2 × 2 table in the standard manner. Agreement between macroscopic and histologic diagnoses at each tissue site was measured using the Cohen’s kappa coefficient, a measure of agreement for categorical variables.

## RESULTS

One-hundred forty patients were identified and reviewed for possible inclusion in the study; 61.4% were female and mean age at biopsy was 9.1 years (Table 1). The most frequent presenting symptoms were abdominal pain, constipation, bloating, fatigue, and irritability; 10% of patients were asymptomatic (Table 1). The mean aTTG prior to biopsy was 393.9 with a range of 0.9 to 3550 (Table 1). In this population, esophageal biopsies were taken in 54.3% of patients (*n* = 76), and gastric biopsies were taken in 77.9% (*n* = 109). Of the patients with esophageal or gastric biopsies, five pediatric gastroenterologists performed upper endoscopy (gastroenterologist #1 - 40 patients, #2 - 33 patients, #3 - 19 patients, #4 - 13 patients, #5 - 9 patients) and three pathologists interpreted the specimens (pathologist #1 - 70 patients, #2 - 43 patients, #3 - 1 patient).

**Table 1:** Characteristics of pediatric celiac disease patients reviewed for study inclusion at the time of upper endoscopy and biopsy (n = 140) n (%)

	**Result**
Demographics	
Female gender	86/140 (61.4)
Age at biopsy (yr) (mean ± SD)	9.1 ± 4.3
Weight at biopsy (kg) (mean ± SD)	34.7 ± 18.9
Height at biopsy (cm) (mean ± SD)	134.4 ± 25.1
Presenting symptoms	
Abdominal pain	87/140 (62.1)
Constipation	41/140 (29.3)
Bloating	40/140 (28.6)
Fatigue	37/140 (26.4)
Irritability	36/140 (25.7)
Poor weight gain	35/140 (25.0)
Diarrhea	33/140 (23.6)
Vomiting	13/140 (9.3)
Asymptomatic	14/140 (10.0)
Serology	
aTTG (*n* = 137) (mean ± SD)	393.9 ± 634.1 (0.9-3550)
IgA (*n* = 75) (mean ± SD)	1.3 ± 0.7 (0.1-3.1)

aTTG: Anti-tissue transglutaminase; IgA: Immunoglobulin A.

**Figure 1:**
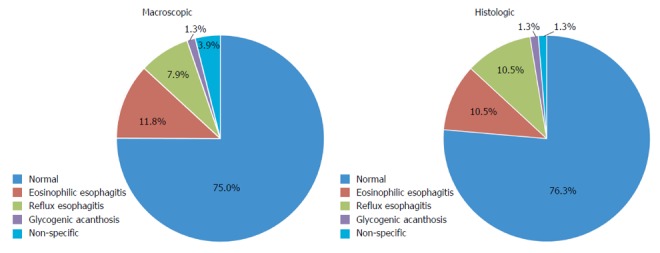
Macroscopic and histologic findings in the esophagus of pediatric patients undergoing upper endoscopy for investigation of celiac disease.

A normal macroscopic appearing esophagus was reported in 75.0% (57/76) of patients (Table 2). All these 57 patients with normal macroscopic esophageal appearance had normal histology, except for two with eosinophilic esophagitis and five with reflux esophagitis. Macroscopic abnormalities visible at upper endoscopy included: eosinophilic esophagitis (9/76, 11.8%), reflux esophagitis (6/76, 7.9%), glycogenic acanthosis (1/76, 1.3%) and non-specific abnormalities (3/76, 3.9%) (Figure 1). These non-specific macroscopic abnormalities were nodularity and a prominent gastro-esophageal Z-line. Endoscopic signs of eosinophilic esophagitis included exudates, trachealization and linear furrowing. Macroscopic signs of reflux esophagitis were erythema and friability, erosions and ulceration. Esophageal biopsies revealed normal histology in 76.3% (58/76) of patients. Histologic esophageal abnormalities reported included: eosinophilic esophagitis (8/76, 10.5%), reflux esophagitis (8/76, 10.5%), glycogenic acanthosis (1/76, 1.3%) and non-specific abnormalities (1/76, 1.3%). The latter was increased eosinophils at the gastro-esophageal junction.

**Table 2:** Macroscopic and histologic findings in the esophagus of pediatric patients undergoing upper endoscopy for investigation of celiac disease n (%)

**Diagnosis**	**Endoscopic**	**Histologic**
Normal	57/76 (75.0)	58/76 (76.3)
Eosinophilic esophagitis	9/76 (11.8)	8/76 (10.5)
Reflux esophagitis	6/76 (7.9)	8/76 (10.5)
Glycogenic acanthosis	1/76 (1.3)	1/76 (1.3)
Non-specific abnormalities	3/76 (3.9)	1/76 (1.3)

**Figure 2:**
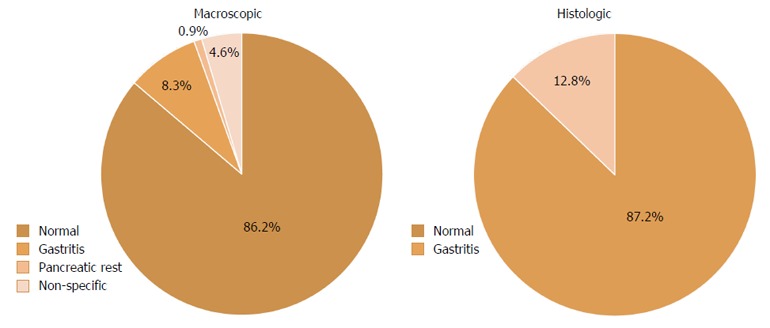
Macroscopic and histologic findings in the stomach of pediatric patients undergoing upper endoscopy for investigation of celiac disease.

In the stomach, 86.2% (94/109) of patients had a normal macroscopic appearance (Table 3), while macroscopic abnormalities included gastritis (9/109, 8.3%), pancreatic rest (1/109, 0.9%) and non-specific abnormalities (5/109, 4.6%) (Figure 2). Endoscopic signs of gastritis described included edema, hyperemia and erosions. The non-specific gastric abnormalities reported were non-specific erythema, edema and hyperemia. Biopsies of the stomach were normal in 87.2% (95/109) of patients. Gastritis was found in 12.8% (14/109) of gastric specimens, with only one child having proven *H. pylori* infection. Only 11/94 patients with a normal endoscopic gastric appearance had a histological diagnosis of gastritis. The patient with *H. pylori* infection had a macroscopic diagnosis of gastritis.

**Table 3:** Macroscopic and histologic findings in the stomach of pediatric patients undergoing upper endoscopy for investigation of celiac disease n (%)

**Diagnosis**	**Endoscopic**	**Histologic**
Normal	94/109 (86.2)	95/109 (87.2)
Gastritis	9/109 (8.3)	14/109 (12.8)
Pancreatic rest	1/109 (0.9)	-
Non-specific abnormalities	5/109 (4.6)	-

Of the 87 patients who reported abdominal pain as a presenting symptom, 75.9% had normal macroscopic appearance of both the esophagus and stomach. Seventy-two of these 87 patients had biopsies taken from at least one of the esophagus or stomach, and 70.8% of these patients had normal histologic results. Eosinophilic esophagitis was found in 4 patients reporting abdominal pain, by both macroscopic and histologic diagnosis. Esophagitis was found histologically in 6 of these patients, 1 of which was associated with macroscopic changes. Gastritis was found histologically in 8 patients with abdominal pain, 1 of which was associated with macroscopic abnormalities. Sixteen of the patients with abdominal pain had a normal appearing esophagus and stomach by endoscopy and had no biopsy taken from either site due to gastroenterologist practice. The other gastrointestinal presenting symptoms relevant to the esophagus and stomach reported in the study population were bloating and vomiting. Of the 40 patients who reported bloating, 82.5% received a normal macroscopic diagnosis in the esophagus and stomach; 71.9% of this group who had biopsies taken from at least one of these sites (*n* = 32) also had normal histologic results. In the 13 patients with vomiting, 61.5% had normal esophageal and gastric macroscopic diagnoses and 63.6% of the 11 patients who had biopsies taken also had normal histology.

The sensitivity of normal macroscopic appearance for predicting normal esophageal histology was 86.2% (97.5%CI: 74.6%-93.9%). The sensitivity of normal macroscopic appearance for predicting normal gastric histology was 87.4% (97.5%CI: 79.0%-93.3%). The positive predictive value of normal macroscopic diagnosis for normal histology in the esophagus and stomach were 87.7% and 88.3%, respectively. The Cohen’s kappa coefficient representing agreement between esophageal macroscopic and histologic diagnoses for all of the gastroenterologists as a group was 0.464 (95%CI: 0.233-0.695, *P* < 0.001), and in the stomach was 0.085 (95%CI: -0.133-0.303, *P* = 0.372).

## DISCUSSION

The findings of this study suggest that a normal macroscopic appearance in the esophagus and stomach is adequate to predict normal tissue histology in patients undergoing endoscopy for the diagnosis of pediatric celiac disease, obviating the need for routine biopsies from these additional gastrointestinal sites. Limiting the number of tissue biopsies may potentially reduce endoscopy time and costs, as well as endoscopy-associated risks such as bleeding or perforation. In Alberta, based on estimates from the Northern Alberta Clinical Trials and Research Centre, the estimated costs associated with processing each gastrointestinal biopsy specimen is $48, plus $55 for pathologist reporting[13]. Reducing the number of total routine biopsies during endoscopy for CD, and hence costs as well as shortened total endoscopy times, could have important system-level implications.

There is a relative paucity of recent studies in the literature investigating the sensitivity of macroscopic diagnosis for predicting tissue histology in pediatrics, particularly in patients with celiac disease. Several adult studies done prior to 1990 reported the correlation between abnormal gastric macroscopic and histologic findings in adults with dyspepsia to be about 50%[14,15]. Black et al[16] studied the sensitivity of endoscopic appearance for predicting abnormal gastric and duodenal histology in pediatric patients with a variety of gastrointestinal complaints in 1988, finding higher severity of reported endoscopic disease as compared to histologic results. Dahshan and Rabah[17] published a similar study in 2000 evaluating the correlation between esophageal and gastric endoscopic and histologic findings in 204 children. They reported sensitivities of 81% in the esophagus and 86% in the stomach, and esophageal and gastric specificities of 41% and 43%, respectively[17]. Long et al[18] reported a sensitivity of 54% and specificity of 92% for prediction of duodenal histology by macroscopic findings in pediatric biopsy-proven duodenitis. A recent article investigated duodenitis in children with multiple gastrointestinal diagnoses, including CD, and found a sensitivity of 34% for endoscopic diagnosis in predicting duodenal histology[19]. Lastly, Sheiko et al[20] reported the correlation of macroscopic and histologic findings in 1000 pediatric patients with a variety of gastrointestinal concerns, 6.6% of who had histology consistent with celiac disease. This group reported a kappa coefficient of 0.45 for endoscopic and histologic concordance in the esophagus, and 0.18 for gastric concordance, recommending routine collection of esophageal, gastric and duodenal specimens in their broad, mostly non-celiac population[20]. Significant improvements in endoscopic imaging capabilities and detail have been made over the past few decades, and this remains the first pediatric study to specifically investigate the ability of normal macroscopic findings to predict normal esophageal and gastric histology in patients with celiac disease.

While literature focused on coexistent gastrointestinal diagnoses at endoscopy in pediatric CD is limited, the coexistent esophageal diagnoses found in this study, specifically eosinophilic esophagitis and gastritis, are similar to that which has been previously described. Stewart et al[21] reported concomitant eosinophilic esophagitis in 3/245 patients diagnosed with CD, with a standardized incidence ratio of 48.4 in their population. In a case series of 17 children with eosinophilic esophagitis, 6/17 were also found to have CD though three of these patients had subsequent histologic remission on a gluten free diet[22]. The authors concluded that the eosinophilic infiltration in these patients may have been directly related to the CD itself[20]. A large Italian study of 230 pediatric CD patients found esophagitis in 12.6% of their study population, which is a very similar prevalence to the current study[23]. Both chronic superficial and lymphocytic gastritis have been described in pediatric CD, and it has been suggested that mucosal involvement in CD is not limited to the duodenum[24]. Oderda et al[23] reported chronic superficial gastritis in 40% of their patients. Another recent group described gastritis in a group of children with CD, finding chronic superficial gastritis in 21% of patients, lymphocytic gastritis in 7%, interstitial gastritis in 0.5% and *H. pylori*-related gastritis in 2.7%[24]. In this study, lymphocytic gastritis was seen predominantly in the children with the most advanced CD on duodenal biopsy (Marsh grade 3C) and seemed to correlate with longer exposure to gluten[24]. The authors concluded that “gastric intraepithelial lymphocytosis may represent a concurrent manifestation of CD rather than a separate entity in the pediatric population[24]”. Chronic superficial gastritis was more prevalent in children with gastrointestinal symptoms as opposed to an atypical presentation of CD and showed improvement with gluten free diet; it was theorized to be due to CD-related delayed gastric emptying[24]. Interestingly, only 20.8% of the children with chronic superficial gastritis had macroscopic abnormalities[24].

Regardless, in this study we found few additional diagnoses that would alter clinical management and were not identified macroscopically. Two patients with histologic eosinophilic esophagitis and five with reflux esophagitis had normal appearing endoscopy, however the clinical significance of these incidental microscopic changes is unclear and may not necessitate any changes in patient management. In the stomach, 11 out of 94 patients with histologic gastritis had a normal endoscopic diagnosis. The singular case of *H. pylori*-related gastritis was correctly identified by endoscopy. Clinical presenting symptoms were not reliable predictors of macroscopic or histologic abnormalities. Overall, this study supports the conclusion that, in the absence of macroscopic abnormalities, few clinically significant gastric and esophageal diagnoses would be missed without biopsies from these sites.

Advances in serological testing have shifted the focus of celiac disease diagnosis largely to serology results, and biopsy is being thought of increasingly as confirmatory. Current European guidelines propose that pediatric patients with classic symptoms of celiac disease, at risk HLA genes and markedly elevated aTTG antibodies (greater than ten times the upper limit of normal) may not require intestinal biopsy for diagnosis. Yet this approach has not been widely adopted in North America. There are likely to be multiple reasons for lack of acceptability of a serological diagnostic approach in North America, including fear of inaccurately committing patients to a lifelong gluten free diet and lack of standardization of celiac serology tests between laboratories[1]. The concern has also been expressed that coexistent diagnoses might well be missed by a biopsy avoiding approach[7]. This study would suggest that this a minimal concern and should not preclude considering a serological diagnostic approach in North America, which would have significant resource and system implications[5]. Hence, this study is important as it serves as the first pediatric study to focus specifically on esophageal and gastric coexistent diagnoses in CD, helping to address one barrier to the adoption of a serological approach to CD diagnosis in Canada.

This study has a few limitations. First, as this was a retrospective review, multiple practitioners were involved in performing endoscopy and in interpreting histological specimens, so variations in interpretation and diagnosis between individuals may have occurred. The gastroenterologists and pathologists were also not blinded to the patients’ clinical history, which could also have influenced their interpretation of endoscopic and histologic findings. Additionally, there were an unequal number of patients studied who had esophageal biopsies taken as compared to those with gastric biopsies (76 *vs* 109 patients, respectively). This of itself suggests that endoscopists may already attempt to reduce the number of biopsies taken based on macroscopic findings and their own perceptions of the relevance of any biopsy findings in that setting. Unfortunately, this also biases our comparison between groups in terms of whether the diagnostic performance of macroscopic appearance for histology is more accurate in one intestinal site compared to the other. Nevertheless, this study provides evidence to support the current approach of those endoscopists who do not take routine gastric and/or esophageal biopsies in the setting of CD diagnosis. It also provides evidence to improve efficiency and reduce cost in current endoscopic and biopsy practices for pediatric CD by avoiding routine esophageal and gastric biopsy approach in the absence of endoscopic findings.

In conclusion, the findings of this study suggest that normal macroscopic diagnosis is strongly predictive of normal histology in the esophagus and stomach in pediatric patients with CD. Therefore, routine esophageal and gastric biopsies during endoscopy for pediatric CD are not required, in the absence of gross macroscopic findings. The implication of limiting biopsies to the duodenum and duodenal bulb may be both cost and time-saving. Additionally, we report a prevalence of coexistent gastrointestinal findings to CD in our patient population of 21% in the esophagus (including eosinophilic esophagitis and reflux esophagitis) and 13% with gastritis. However, the prevalence of clinically relevant diagnoses that may be missed by a biopsy-avoiding approach in the absence of macroscopic abnormalities is very low. Overall, the results of this study have the ability to promote standardization and optimal resource allocation for routine diagnostic practices for pediatric CD.

## ACKNOWLEDGMENTS

The authors would like to thank Maryna Yaskina and Sung Hyun Kang from the Women and Children’s Health Research Institute for their assistance with statistical analysis. This research has been funded by the generous support of the Stollery Children's Hospital Foundation through the Women and Children’s Health Research Institute.

## COMMENTS

### Background

Celiac disease (CD) is the most common autoimmune enteropathy in children. In North America, diagnosis requires upper endoscopy and duodenal biopsies. Recent guidelines support diagnosis without biopsy in select pediatric patients, yet concerns exist over the potential for missing alternate tissue diagnoses. Additionally, in the absence of specific guidelines, practice is likely to vary regarding additional biopsies taken during endoscopy, which may be taken routinely from sites other than the duodenum even in the face of normal microscopic findings. Additional biopsies have the potential to add considerable additional cost for the pathological assessment of this common duodenal disorder.

### Research frontiers

There is a paucity of studies investigating the frequency of endoscopic and histological abnormalities in intestinal sites apart from the duodenum in pediatric patients with CD. Additionally, the utility of endoscopic diagnosis in predicting tissue histology in the stomach and esophagus in CD has yet to be studied.

### Innovations and breakthroughs

The findings of this study suggest that normal macroscopic diagnosis is strongly predictive of normal histology in the esophagus and stomach in pediatric patients with celiac disease. Therefore, routine esophageal and gastric biopsies during endoscopy for pediatric celiac disease are not required in the absence of gross macroscopic findings.

### Applications

The implication of limiting biopsies to the duodenum and duodenal bulb may be both cost and time-saving. Overall, the results of this study have the ability to promote standardization and optimal resource allocation for routine diagnostic practices for pediatric celiac disease.

### Terminology

Macroscopic - visual appearance of gastrointestinal mucosa at the time of endoscopy. Histologic - microscopic appearance of gastrointestinal mucosa cells.

### Peer-review

This is a well designed, performed and written clinical retrospective study for the determination of the sensitivity of normal esophageal and gastric macroscopic appearance in predicting normal tissue histology at sites other than the duodenum in pediatric CD patients. They concluded that routine esophageal and gastric biopsies during endoscopy for pediatric CD are not required, in the absence of macroscopic abnormalities.
